# Evaluation of Regional Variability and Measurement Reproducibility of Intravoxel Incoherent Motion Diffusion Weighted Imaging Using a Cardiac Stationary Phase Based ECG Trigger Method

**DOI:** 10.1155/2018/4604218

**Published:** 2018-04-17

**Authors:** Zhiming Xiang, Zhu Ai, Jianke Liang, Guijin Li, Xiaolei Zhu, Xu Yan

**Affiliations:** ^1^Department of Radiology, Guangzhou Panyu Center Hospital, Medical Imaging Institute of Panyu, 8 Fuyu Dong Road, Panyu District, Guangzhou 510080, China; ^2^Siemens Healthcare, MR Scientific Marketing NE Asia, Guangzhou, China; ^3^Siemens Healthcare, MR Collaboration NE Asia, Shanghai, China

## Abstract

**Purpose:**

To evaluate the performance of an optimized ECG trigger diffusion weighted imaging (DWI) sequence in liver and its application in liver disease.

**Materials and Methods:**

Eighteen healthy volunteers underwent intravoxel incoherent motion diffusion weighted imaging (IVIM-DWI) scan of the liver twice in 1.5T MR scanner with signed informed consent approved by local ethic committees. A new method, called cardiac stationary phase based ECG trigger (CaspECG), and FB method were applied. The apparent diffusion coefficient (ADC) and the IVIM parameters, including pure diffusion coefficient (*D*), perfusion-related diffusion coefficient (*D*^⁎^), and perfusion fraction, (PF) were calculated, and then 18 region of interests were drawn on these parameter maps independently by two readers through whole hepatic lobe. The regional variability and reproducibility between two repeated scans were evaluated using interclass correlation coefficients (ICCs) and Bland-Altman plot, respectively, and compared between the CaspECG and FB methods. The signal-to-noise ratio (SNR) of DWI data was also evaluated.

**Result:**

Compared to the FB method, the proposed CaspECG method showed significant higher SNRs in DWI data, lower regional variability between left and right hepatic lobes, and higher reproducibility of ADC, PF, D, and D^⁎^ between repeat scans [left lobe, limit of agreement (LOA) of Bland-Altman plot: 10.1%, 18.3%, 19.8%, and 59.2%; right lobe, LOA: 10.25%, 14.15%, 16.45%, and 39.45%]. D^⁎^ showed the worst reproducibility in all parameters.

**Conclusion:**

The novel CaspECG method outperformed the FB method in compensating the cardiac motion induced artifacts in DWI data and generating more reliable quantitative parameters, with less regional variability and higher repeatability, especially in the left hepatic lobe.

## 1. Introduction

Diffusion weighted imaging (DWI) has been widely used in the diagnosis, prognosis, and evaluation of liver diseases [1–4]. But the apparent diffusion coefficient (ADC) which used traditional mono-exponential model could not fully account for the microcirculation and perfusion effect of liver tissue [5, 6]. The intravoxel incoherent motion (IVIM) method uses a biexponential model first proposed in the late 1980s and was used to quantify simultaneously the tissues diffusion and blood perfusion components [7]. Three parameters can be simultaneously acquired from this model: the real diffusion coefficient (D) of tissue, perfusion-related diffusion coefficient (D^*∗*^), also called pseudo-diffusion, and the perfusion fraction (PF) of the total signal [7, 8]. Previously studies have shown that IVIM is helpful in clinical diagnosis and treatment assessment of liver disease [9–15].

The quality ADC and IVIM parameters could be influenced by many factors, such as physiological motion and corporation of patient. Many earlier studies have reported that physiological motion, such as respiratory and cardiac, may cause signal loss in the liver DWI image and measurement error of ADC, especially in left lobe of liver [16]. Some other researchers reported that regional variability of ADC in liver [17] became more obvious when close to the heart area.

Previously researches reported that some respiration-trigger techniques and electrocardiography (ECG) trigger method could compensate the influence of respiratory and cardiac motion [3, 16]. Recently, studies showed that ECG trigger method outperformed respiration-trigger method in generating more robust ADC maps [18, 19], also reducing measurement errors or regional variability of IVIM parameters [18–20]. In these studies, a fixed ECG trigger delay time and TR were mainly applied for all patients with different heart rate, while its performance may not be stable because the relative stationary phase of heart cannot be defined by heart rate alone. In addition, the influence of the ECG trigger method in diagnosing liver disease was not fully investigated.

In this study, an optimized ECG trigger method was applied, which modulates ECG trigger time based on cardiac relative stationary phase (CaspECG). We applied the optimized method and conventional free-breathing method in IVIM imaging and compared their image quality, SNR, reproducibility of quantitative parameters, and most importantly the diagnosis accuracy in several liver diseases.

## 2. Materials and Methods

### 2.1. CaspECG Technique

**Figure 1:**
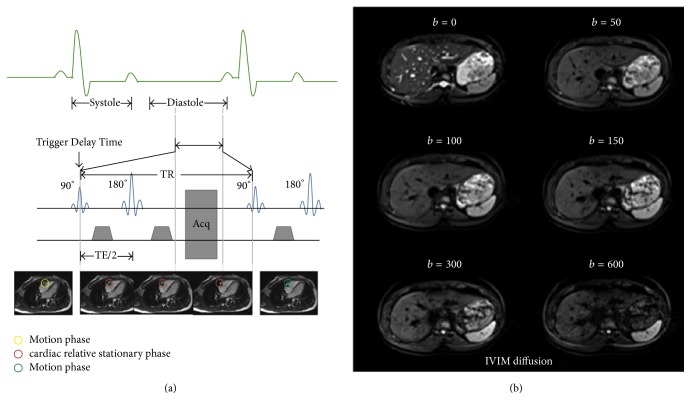
(a) Schematic of the proposed cardiac stationary phase based ECG trigger (CapsECG) technique. The yellow circles represented the motion phase before the cardiac relative stationary, the red circles represented the cardiac relative stationary phase, and the green circles represented the motion phase after the cardiac relative stationary. The trigger delay time and data acquisition window were decided according to each subject's cardiac relative stationary states, making sure images were collected during ventricular diastolic phases. (b) Intravoxel incoherent motion diffusion MR in normal liver lobe by CapsECG technique with *b* = 0, 50, 100, 150, 300, and 600 sec/mm^2^.

A paradigm of the CaspECG technique was described as follows (Figure 1): a 2D high temporal resolution steady-state-free-precession 4 chamber heart cine imaging was fast applied with breath-hold (9 ms, flip angle: 180°; thickness 6 mm; 1 phase). The trigger delay time and the sampling duration time were determined by identifying rest periods of standard apical four chamber views [21]: the relative rest period of right coronary artery was carefully observed layer by layer after the 4-chamber heart cinema was finished and was defined as the period when the coronary artery position was fixed and the shape of this blood vessel remained unchanged. This specific period was taken into account for the cardiac relative stationary phase, in which it might belong to the diastolic phases of left ventricular [22]. The beginning of cardiac relative stationary states was set as the trigger delay time, and the time interval of the cardiac relative stationary was set as the sampling duration time (TR); finally the detailed times were set into the SS-EPI sequences for IVIM-DWI protocol.

### 2.2. Study Population

The whole prospective study was approved by institutional review board of Guangzhou Panyu Center Hospital, with written informed consent from all participants before enrolment. A total of 18 volunteers (9 men and 9 women, age range: 19 to 36 years, mean age, 24.10 ± 2.98 years) were selected from May 2016 to August 2016, and IVIM-DWI was performed on each volunteer after informed consent was obtained. The inclusion criteria for this study were as follows: (a) no history of liver disease (including mild fatty liver) and viral hepatitis-associated serological markers were negative; (b) no medications taken to damage liver function within six months; and (c) no history of liver surgery or alcohol abuse. The exclusion criteria included (a) MRI contraindications such as in vivo metal implants, claustrophobia, and cardiac pacemaker implantation; (b) not taking medicine in the near future; (c) the subjects who during scan had emotional tension or could not tolerate prolonged examination and who failed to complete the examination; and (d) poor image quality that was insufficient for image analysis.

### 2.3. MR Imaging Protocol

Heart rates were measured twice by diagram of electrocardiograph monitoring before each protocol of MR exams. All volunteers were examined with a 1.5T MR scanner (MAGNETOM Avanto; Siemens Healthcare, Erlangen, Germany). A combination of 6-channel body coil and 6-channel spine coil were used to cover the subject's whole upper abdomen.

All volunteers underwent IVIM-DWI of the liver twice on the same day by using both the FB and CaspECG technique. Each volunteer was moved outside the scanner and relocate again between two IVIM-DWI scans. The time interval between two sessions was approximately 20 minutes. The technical methods were as follows: 4-chamber heart cinema with FOV = 340 × 276 mm, TR = 45.9 ms, TE = 1.28 ms, average = 1, slice thickness = 6 mm, and TA = 9 s. CaspECG IVIM-DWI sequences were acquired with 6*b* values (0, 50, 100, 150, 300, and 600 sec/mm2), with the following parameters: FOV = 400 × 262 mm, TR = 110 ms, TE = 66 ms, matrix = 128 × 128, average = 2, concatenation = 5, slices = 5, slice thickness = 5 mm, 3-scan-trace mode, and TA = 9.40 min, while the free-breathing DWI sequence were acquired with the same *b* value and parameters except for concatenation = 1, TR = 2200 ms, and TE = 66 ms, without ECG trigger.

### 2.4. Image Postprocessing

The ADC was calculated with linear least-squares fitting of all the six *b* value data on a pixel-by-pixel basis according to the mono-exponential diffusion equation [16]:

(1) $$\begin{array}{c} ln\left(\;{SI}_{b}\;\right)=-b\cdot ADC+\ln\left(\;{SI}_{0}\;\right), \end{array}$$

where SI_*b*_ is the signal intensity at a given *b* value and *S*_0_ is the signal intensity for *b* = 0 sec/mm^2^.

For IVIM-based analysis, the IVIM-DWI data from both FB and CapsECG acquisitions were fitted by the biexponential diffusion equation [5]:

(2) $$\begin{array}{c} S_{b}=S_{0}\left(\;f\times e^{-{bD}^{*}}+\left(\;1-f\;\right)\times e^{-bD}\;\right). \end{array}$$

A segmented fitting algorithm was adopted here using in-house developed software (MATLAB R2012b, Mathwork Software). The fitting method first assumed the perfusion component can be neglect for diffusion data with *b*⩾200, and then the diffusion coefficient (*D*) and *S*_0_′ can be calculated using mono-exponential equation *S*_*b*_ = *S*_0_′ exp (−*b* × *D*). The perfusion fraction (PF) is calculated by PF = 1 − *S*_0_′/*S*_0_. Then the known *D* and PF were applied in IVIM equation and pseudo diffusion factor *D*^*∗*^ were then calculated again using mono-exponential equation. In addition, the biexponential diffusion decay curve at the locations of left and right lobe was computed using the estimated IVIM parameters from both two techniques.

### 2.5. Data Analysis

Firstly, we quantitatively compared the image quality of DWI data between Casp-ECG and FB method using the signal-to-noise ratio (SNR) calculated by following equation:

(3) $$\begin{array}{c} SNR=\frac{{SI}_{liver}}{{SD}_{background}}, \end{array}$$

where the signal intensity of liver (SI_liver_) and standard deviation of background signal (SD_background_) were measured. Three 100 mm^2^ circular regions of interest (ROIs) were selected in liver parenchyma to obtain liver signal intensity (SI), and three 100 mm^2^ circular ROIs were selected in the extra-abdominal to obtain background standard deviation (SD).

Second, a region-of-interest (ROI) analysis was applied to evaluate the reproducibility and region variability of quantitative parameters. The ROIs were drawn with ImageJ software (National Institutes of Health, Bethesda, MD) by two readers working independently. For each volunteer, 18 circular ROIs were positioned in the liver with a fixed size of 100 mm^2^ on DWI baseline data (*b* = 0) and then were applied to all parameter maps. For each scan, three slices were selected according to image quality, and then in each slice three ROIs were drawn on left and right hepatic lobe separately (large intrahepatic vessels and prominent artifacts were excluded). All the 18 ROIs (9 ROIs each lobe) were evenly distributed in the left and right hepatic lobe through the whole lobe, and they were placed in locations as similar as possible among two repeat scans and two techniques.

### 2.6. Statistics

In ROI analysis, (1) the regional variability of IVIM-DWI quantitative parameters in left and right lobes was separately evaluated by interobserver agreement of ADC, PF, *D*, and *D*^*∗*^ values measured from all 9 ROIs. The interclass correlation coefficient (ICCs) was used here based on measurements from two observers. An ICC greater than 0.75 was defined as an indicator of good agreement [23]; (2) the regional variability between the right and left hepatic lobes was additionally evaluated for two techniques and compared using the paired *t*-test, where the mean values of quantitative parameters in left and right lobe were acquired by averaging the ROIs in corresponding lobe; and (3) the reproducibility of quantitative parameters was evaluated between 2 repetition scans with the Bland-Altman method [24]. The mean absolute difference (bias) and the 95% confidence interval of the mean difference (limits of agreement [LOAs]) between the first and second IVIM-DWI data were compared [25].

Statistical analyses were performed by using SPSS (version 19.0; SPSS, Chicago, Ill) and MedCalc (MedCalc, Mariakerke, Belgium) software. All reported *P* values were two sided. Differences were considered significant when *P* values were less than 0.05.

## 3. Results

### 3.1. SNR Analysis

CaspECG showed better image quality than FB in IVIM-DWI data, with significant higher SNRs (for *b* = 600 sec/mm^2^, CaspECG technique: SNR = 151.60 ± 83.19; FB technique: SNR = 58.46 ± 51.13; *P* < 0.001) in hepatic lobe (Table 1). Generally, the SNR of DWI data decrease as *b* value increases, except for *b* = 0 sec/mm^2^, where lower average number was applied (3-scan-trace mode were used for nonzero *b* value).

**Figure 2:**
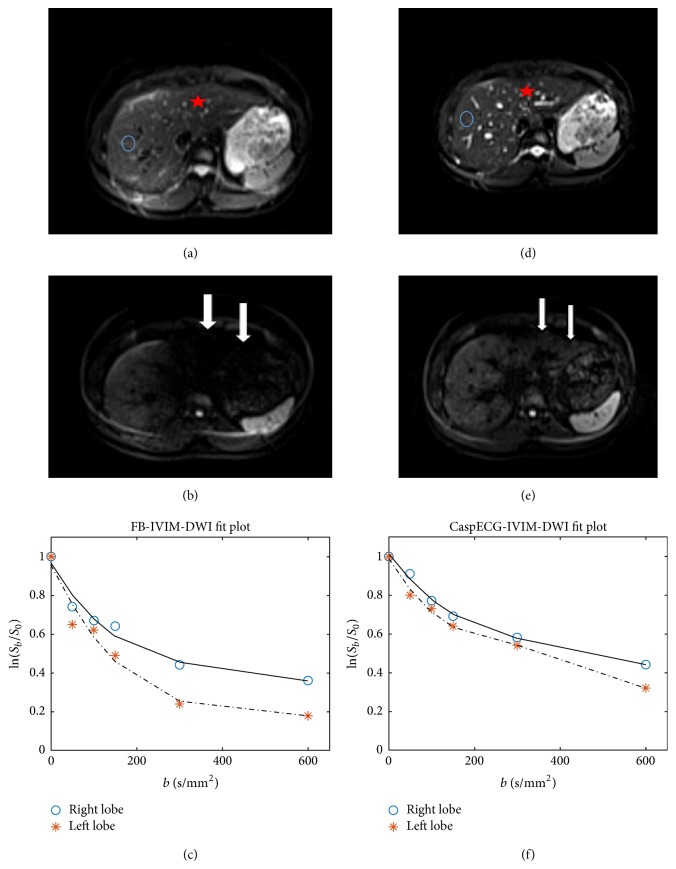
Comparison of free-breathing (FB) and CaspECG techniques on DWI data from a healthy 22-year-old man. DWI data with *b* = 0 (a, d) and 600 (b, e) sec/mm^2^ were shown and compared ((a, b, c) FB method, (d, e, f) CaspECG metod). The signal decay and biexponential fitting curves were also shown (c, f). The blue circles in (a, d) and red snowflakes (b, e) denote right and left hepatic lobes, respectively. In DWI data at *b* = 600 sec/mm^2^ (b), significant signal loss was found in the left hepatic lobe (arrows) for FB method, but not for CaspECG method, which influenced the following IVIM fitting (c).

Moreover, the attenuation curves of diffusion signal from both two techniques were shown and fitted by IVIM model (Figure 2). The result showed that the CaspECG technique provided higher consistency of biexponential attenuation curve between the left and right hepatic lobes.

### 3.2. Regional Variability Analysis of 9 ROIs in Each Hepatic Lobe

Interobserver agreement of ADC, PF, *D*, and *D*^*∗*^ were evaluated in left and right lobes separately (9 ROIs each lobe) using ICCs (Table 2). For ADC values measurements, the highest ICC was 0.988, and it came from the left hepatic lobe with CaspECG IVIM-DWI. The highest ICC was 0.951 and 0.946 for PF and *D* values measurements, respectively. In the entire interobserver agreement test, the *D*^*∗*^ showed consistently lower ICC than the other parameters. Both two techniques showed low regional variability for all the quantitative parameters.

### 3.3. Regional Variability Analysis between Left and Right Hepatic Lobes

**Figure 3:**
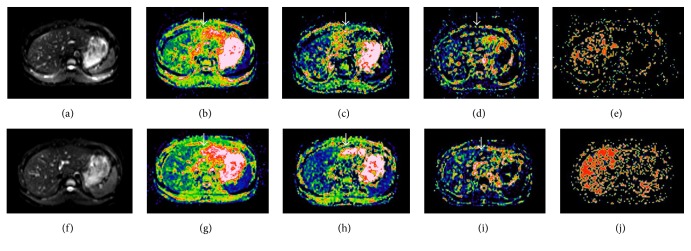
Parametric IVIM maps obtained by using the FB IVIM-DWI (a–e) and CapsECG IVIM-DWI (f–j) techniques. The DWI baseline data, ADC, *D*, PF, and *D*^*∗*^ map were shown from left to right for both two techniques. The ADC, *D*, and PF maps show significant difference between left and right hepatic lobe (arrows) for FB method, while the difference was less significant for CaspECG method. For both two methods, the *D*^*∗*^ maps appear heterogeneous and coarse, indicating a large variability in *D*^*∗*^ values in the liver.

The ROI measurements of ADC and IVIM parameters from both FB and CaspECG techniques were summarized in Table 3. Although both techniques showed significant differences of ADC, *D*, and *D*^*∗*^ values between left and right hepatic lobe, the CaspECG method showed less absolute differences. In addition, PF showed significant differences between left and right hepatic lobe only for FB technique (*P* < 0.001), but not for CaspECG (Figure 3).

Meanwhile, compared to FB method, the CaspECG technique generated lower ADC, PF, *D*, and *D*^*∗*^ values especially for left hepatic lobe, with significant differences found for ADC and PF in both left and right hepatic lobe, and *D* only in the left hepatic lobe (*P* < 0.05).

### 3.4. Reproducibility Analysis

**Figure 4:**
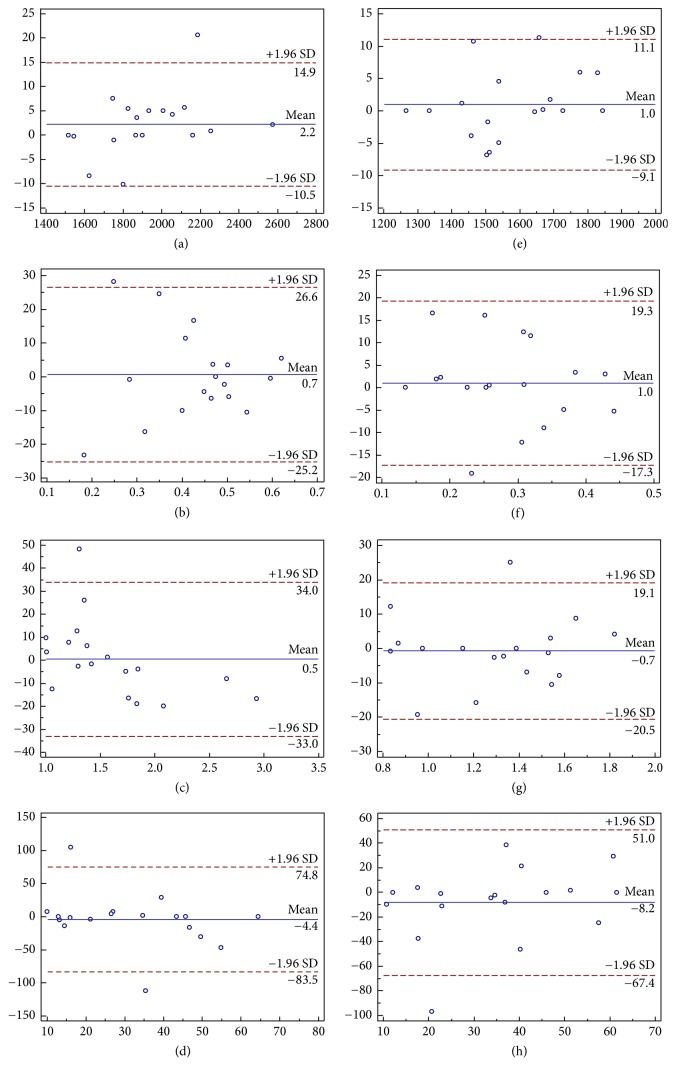
The Bland-Altman plots of ADC, PF, *D*, and *D*^*∗*^ (from upper to bottom) in left hepatic lobe with FB IVIM-DWI (left) and CaspECG (right) IVIM-DWI. Blue line = mean absolute difference and red lines = 95% confidence interval of the mean difference (LOA). The CaspECG method showed higher reproducibility in all quantitative parameters than FB method with lower LOA.

**Figure 5:**
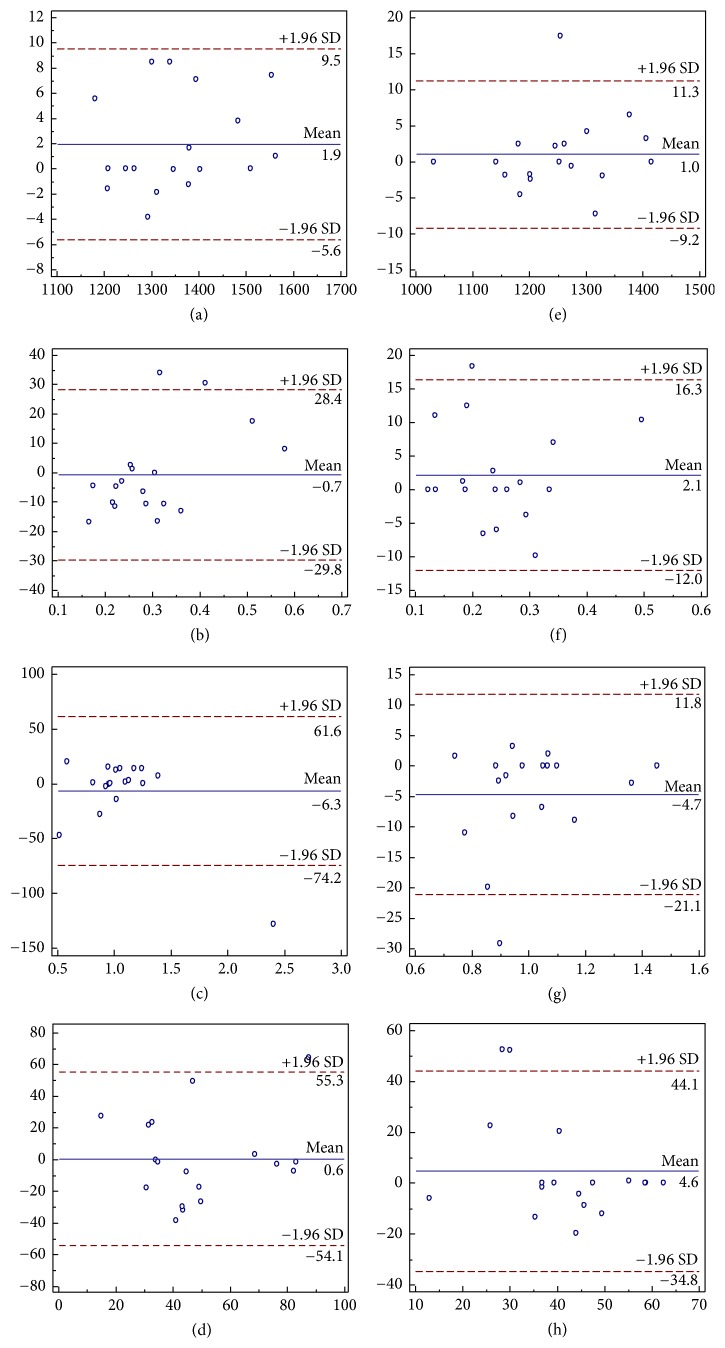
The Bland-Altman plots of ADC, PF, *D*, and *D*^*∗*^ (from upper to bottom) in right hepatic lobe with FB IVIM-DWI (left) and CaspECG (right) IVIM-DWI. Again, the CaspECG method showed higher reproducibility than FB method.

The Bland-Altman analysis showed that the CaspECG method provided more reproducible quantitative parameter than FB method with lower LOA. In the left hepatic lobe, the LOA of Bland-Altman plot for ADC, PF, *D*, and *D*^*∗*^ were 12.7%, 25.9%, 33.5%, and 79.15% for FB IVIM-DWI, and 10.1%, 18.3%, 19.8%, and 59.2% for CaspECG IVIM-DWI, respectively (Figure 4). In the right hepatic lobe, the LOA of ADC, PF, *D*, and *D*^*∗*^ were 7.55%, 29.1%, 67.9%, and 54.7% with FB, and 10.25%, 14.15%, 16.45%, and 39.45% with CaspECG, respectively (Figure 5).

## 4. Discussion

This study proposed a cardiac stationary phase based ECG trigger (CaspECG) method for IVIM-DWI, which could accurately catch the optimal ECG delay time to minimize the influence of cardiac motion to DWI images. The experiment showed that the CaspECG could significantly improve both quality of DWI data and the quantitative parameters of IVIM in liver, especially for left lobe of liver. Compared to FB method, the CaspECG method generates ADC and IVIM parameters with generally higher consistency in the whole liver, lower regional variability between left and right lobes, and better interscan reproducibility.

As reported in previous study [26], the cardiac motion has more influence on liver DWI than respiration, with signal loss especially at left hepatic lobe. The reason may be due to the nature of diffusion sequence. The cardiac motion is much faster than respiration and could introduce significant movement of tissue during the diffusion encoding phase, which will result in serious signal decay. Thus, in the region influence by cardiac motion such as left hepatic lobe, signal loss of DWI may occur, which may also reduce the accuracy and stability of ADC or IVIM parameters.

The CaspECG method determined the trigger delay time based on the relative stationary phase and coronary morphology of the different subjects. The method of magnetic resonance angiography in coronary artery was used to determine optimal delay time in the study [27, 28], which showed that systolic and early diastolic coronary arteries were the strongest motion, and there was a transient “stay” in the diastolic mid-term [28]. Thus, this method can, to a large extent, ensure that each of the sequence acquisitions fall within the relatively stationary phase of the heart.

The experiment showed that the CaspECG method could significantly minimize the signal loss due to cardiac motion, with better SNR of DWI in whole liver and much less region variability of ADC and IVIM parameters between or within left and right lobe. In FB method, the diffusion coefficient, namely, ADC, *D*, and *D*^*∗*^ values, showed significant differences between left and right hepatic lobe, and the significantly higher value in left lobe may be due to cardiac motion. CaspECG method well compensate the cardiac motion and generate quantitative IVIM parameters with smaller differences between left and right hepatic lobe. This result agreed with previous studies [26, 29–32] that the ECG gating method could better improve the regional variability and measurement reproducibility of ADC than FB or other gating methods.

In addition, we found that, for both the CaspECG and FB method, the IVIM *D*^*∗*^ parameter showed the worst repeatability. Similar to our findings, some previous studies also showed the poor measurement repeatability of *D*^*∗*^ for both liver parenchyma and metastases [26, 33]. Although previous studies have shown that *D*^*∗*^ had more predictable value than traditional ADC [34], due to its low repeatability, special care should be taken to its application.

Although the CaspECG method showed significant improvement for IVIM-DWI quantitative imaging, its acquisition time is much longer than the FB method, and additional effort for ECG monitoring should be applied. Thus, our recommendation is as follows: (1) if the lesion is located in left lobe with significant motion artifacts or a follow-up scan will be applied, ECG trigger method is highly suggested for more accurate and robust diagnosis; (2) in the other case, the FB method could be applied for efficiency.

In this study, our IVIM-DWI scan only covers 5 slices but not the whole liver. The reasons are as follows: (1) the main focus of the work is to evaluate the performance of CaspECG method and compare it to FB method; the whole liver coverage is not necessary; (2) the overall scan time needs control in our study as the ECG-triggering takes long acquisition time and repetition scan was required. In patient study, the whole liver coverage could be achieved by using an efficient *b*-value setting.

Our study had several limitations. First, the volunteers involved in this study were young people, lacking overall representation. Considering the wide range of age distribution of liver MR examination in clinical practice, it might be different from the conclusion of this study. Second, the measurement of ROIs was subjective to the choice of surveyors, which might have subjective influence on the conclusion. Finally, in our study, the selected volunteers were healthy people, lacking analysis and evaluation of different liver diseases, as the microcirculation and cell structure between normal liver and liver lesions are different, and the value of the IVIM parameters is more sensitive to tumor perfusion information. Therefore, this study needs to be further validated in clinical practice.

In conclusion, we evaluate this novel acquisition method which combined DWI sequence with optimized ECG trigger based on cardiac relative stationary phase. The method showed high repeatability and stability of the multiparameters of IVIM-DWI and could be applied to liver disease study in future for validation.

## Figures and Tables

**Table 1:** The SNR of DWI with different *b* values (s/mm^2^) in liver lobe using FB and CapsECG methods.

Technique	*b* = 0	*b* = 50	*b* = 100	*b* = 150	*b* = 300	*b* = 600
FB	127.13 ± 87.32	168.64 ± 114.32	120.19 ± 82.59	118.32 ± 106.16	73.70 ± 53.25	58.46 ± 51.13
CapsECG	342.73 ± 242.47	428.25 ± 284.48	312.32 ± 222.44	288.32 ± 181.62	218.13 ± 112.56	151.60 ± 83.19
*P* value	<0.001					

**Table 2:** The Inter-Observer (Two Observers) Agreement of Quantitative Parameters Measurement from All 9 ROIs in Each Lobe Using Interclass Correlation Coefficients (ICCs).

Agreement	FB				CapsECG			
	Left lobe		Right lobe		Left lobe		Right lobe	
	First examination	Second examination	First examination	Second examination	First examination	Second examination	First examination	Second examination
ADC	0.979 (0.940,0.998)	0.935 (0.854,0.983)	0.981 (0.951,0.993)	0.858 (0.721,0.982)	0.988 (0.971,0.996)	0.905 (0.778,0.985)	0.933 (0.875,0.989)	0.960 (0.894,0.992)
PF	0.926 (0.820,0.972)	0.948 (0.897,0.985)	0.951 (0.870,0.983)	0.887 (0.613,0.971)	0.925 (0.855,0.975)	0.871 (0.695,0.962)	0.947 (0.774,0.990)	0.921 (0.735,0.980)
*D*	0.946 (0.783,0.990)	0.920 (0.648,0.988)	0.905 (0.802,0.961)	0.836 (0.606,0.934)	0.885 (0.751,0.956)	0.889 (0.757,0.954)	0.907 (0.800.0.974)	0.925 (0.731,0.979)
*D* ^*∗*^	0.622 (0.069,0.944)	0.576 (0.121,0.874)	0.869 (0.798,0.941)	0.595 (0.338,0.929)	0.678 (0.454,0.851)	0.448 (0.100,0.748)	0.502 (0.131,0.790)	0.442 (0.044,0.735)

*Note*. —Data in parentheses are 95% confidence intervals.

**Table 3:** Comparison of Quantitative Parameters of Right and Left Hepatic Lobe.

Parameter	FB	CapsECG	*t*	*P*
ADC(×10^−3^ mm^2^/s)				
Left lobe	1.955 ± 0.299	1.598 ± 0.187	5.678	<0.001
Right lobe	1.367 ± 0.126	1.258 ± 0.109	4.026	<0.001
*t*	8.550	8.381		
*P*	<0.001	<0.001		
PF				
Left lobe	0.432 ± 0.117	0.284 ± 0.087	5.787	<0.001
Right lobe	0.304 ± 0.125	0.247 ± 0.094	2.729	0.014
*t*	4.561	1.936		
*P*	<0.001	0.070		
*D*(×10^−3^ mm^2^/s)				
Left lobe	1.580 ± 0.456	1.291 ± 0.315	2.278	0.036
Right lobe	1.001 ± 0.255	0.986 ± 0.194	0.218	0.830
*t*	5.582	3.862		
*P*	<0.001	0.001		
*D* ^*∗*^(×10^−3^ mm^2^/s)				
Left lobe	33.964 ± 18.005	30.111 ± 15.543	0.804	0.433
Right lobe	50.142 ± 24.956	42.259 ± 12.042	1.407	0.177
*t*	3.582	3.011		
*P*	0.002	0.008		

*Note*.-Data are means ± standard deviations, unless indicated otherwise; ^*∗*^*P* values were obtained from the comparison of the parameter values among the two DWI techniques by using the paired *t* test.
